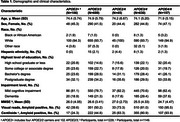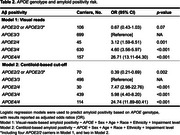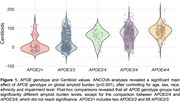# The effect of *APOE* genotype in real‐world clinical settings: results from the IDEAS and ANGI studies

**DOI:** 10.1002/alz70856_097760

**Published:** 2025-12-24

**Authors:** Stefania Pezzoli, Alexis P. Oddi, Ganna Blazhenets, Ale Castruita, Daniel R. Schonhaut, Caroline Jonson, Jhony A. Mejía‐Perez, Kaitlin B Casaletto, Lucy Hanna, Andrew March, Constantine Gatsonis, Maria C. Carrillo, Tatiana M. Foroud, Renaud La Joie, Jennifer S. Yokoyama, Gil D. Rabinovici

**Affiliations:** ^1^ University of California, San Francisco, San Francisco, CA, USA; ^2^ Memory and Aging Center, Weill Institute for Neurosciences, University of California San Francisco, San Francisco, CA, USA; ^3^ Dept. of Biostatistics, Brown University, Providence, RI, USA; ^4^ American College of Radiology, Reston, VA, USA; ^5^ Alzheimer's Association, Chicago, IL, USA; ^6^ Indiana University School of Medicine, Indianapolis, IN, USA

## Abstract

**Background:**

Real‐world data about the clinical utility of *APOE* genotype in memory care are lacking. The Imaging Dementia‐Evidence for Amyloid Scanning (IDEAS) study evaluated the clinical impact of amyloid PET in Medicare beneficiaries with cognitive impairment. The Amyloid Neuroimaging and Genetics Initiative (ANGI) study analyzed DNA from a subset of IDEAS participants. We leveraged this large dataset to assess the predictive value of *APOE* genotype in determining amyloid status and burden in real‐world memory care.

**Method:**

1637 Medicare beneficiaries with cognitive impairment enrolled in ANGI and IDEAS were included. Amyloid PET positivity was determined using (a) local community visual reads and (b) a Centiloid threshold of 24.4. Centiloids were calculated using a PET‐only pipeline in a subset of 1149 participants. Logistic regression assessed the odds of amyloid positivity, while ANCOVA investigated differences in global amyloid burden, measured in Centiloids, adjusting for age, sex, race, ethnicity, and impairment level (mild cognitive impairment or dementia).

**Result:**

Demographics and clinical features are shown in Table 1. Among the total sample, 38% were *APOE4* heterozygotes and 10% were homozygotes. Compared to *APOE3/3*, *APOE3/4* and *APOE4/4* carriers showed higher dementia rates (*p* <0.001) and lower MMSE scores (*p* <0.001). Compared to *APOE3/3*, individuals with the *APOE2/4*, *APOE3/4*, and *APOE4/4* genotypes had higher odds of being amyloid positive, following a dose‐dependent gradient of increasing risk (Table 2). *APOE2* carriers (*APOE2/2* or *APOE2/3*) had lower odds of being amyloid positive compared to *APOE3/3* (significant only with Centiloid threshold, Table 2). A significant interaction between the *APOE3/4* genotype and sex was observed (*p* = 0.004). After adjusting for covariates, females with the *APOE3/4* genotype had 6.77 times higher odds of being amyloid‐positive compared to males with the reference *APOE3/3* genotype (visual reads). Amyloid burden increased progressively across *APOE* genotypes, with higher‐risk genotypes (*APOE3/4* and *APOE4/4*) showing the highest Centiloid values (Figure 1).

**Conclusion:**

These findings are particularly important for real‐world clinical settings, where genetic risk stratification can inform prevention strategies and treatment decisions. Incorporating genetic information into care, including sex‐specific risk assessments, allows for more tailored and personalized interventions, potentially leading to improved clinical outcomes.